# Imaging of pediatric great vessel stents: Computed tomography or magnetic resonance imaging?

**DOI:** 10.1371/journal.pone.0171138

**Published:** 2017-01-31

**Authors:** A. M. den Harder, D. Suchá, R. W. van Hamersvelt, R. P. J. Budde, P. A. de Jong, A. M. R. Schilham, C. Bos, J. M. P. J. Breur, T. Leiner

**Affiliations:** 1 Department of Radiology, Utrecht University Medical Center, Utrecht, The Netherlands; 2 Department of Radiology, Erasmus Medical Center, Rotterdam, The Netherlands; 3 Department of Pediatric Cardiology, Utrecht University Medical Center, Utrecht, The Netherlands; Universitatsklinikum Freiburg, GERMANY

## Abstract

**Background:**

Complications might occur after great vessel stent implantation in children. Therefore follow-up using imaging is warranted.

**Purpose:**

To determine the optimal imaging modality for the assessment of stents used to treat great vessel obstructions in children.

**Material and methods:**

Five different large vessel stents were evaluated in an in-vitro setting. All stents were expanded to the maximal vendor recommended diameter (20mm; n = 4 or 10mm; n = 1), placed in an anthropomorphic chest phantom and imaged with a 256-slice CT-scanner. MRI images were acquired at 1.5T using a multi-slice T_2_-weighted turbo spin echo, an RF-spoiled three-dimensional T_1_-weighted Fast Field Echo and a balanced turbo field echo 3D sequence. Two blinded observers assessed stent lumen visibility (measured diameter/true diameter *100%) in the center and at the outlets of the stent. Reproducibility of diameter measurements was evaluated using the intraclass correlation coefficient for reliability and 95% limits of agreement for agreement analysis.

**Results:**

Median stent lumen visibility was 88 (IQR 86–90)% with CT for all stents at both the center and outlets. With MRI, the T_2_-weighted turbo spin echo sequence was preferred which resulted in 82 (78–84%) stent lumen visibility. Interobserver reliability and agreement was good for both CT (ICC 0.997, mean difference -0.51 [-1.07–0.05] mm) and MRI measurements (ICC 0.951, mean difference -0.05 [-2.52 –-2.41] mm).

**Conclusion:**

Good in-stent lumen visibility was achievable in this in-vitro study with both CT and MRI in different great vessel stents. Overall reliability was good with clinical acceptable limits of agreement for both CT and MRI. However, common conditions such as in-stent stenosis and associated aneurysms were not tested in this in-vitro study, limiting the value of the in-vitro study.

## Introduction

Great vessel stents are commonly implanted in children with congenital heart disease. Complications after stent implantation may include in-stent stenosis and aneurysm formation [1]. Although these complications are relatively rare they require lifelong patient monitoring [2,3]. Computed tomography (CT) is often the preferred imaging modality because it is readily available, rapid, non-invasive and correlates highly with invasive angiography for the detection of stenosis [1]. However, CT is also associated with ionizing radiation which is a well-known concern especially in paediatric imaging as children are more susceptible to the effects of radiation and have a longer expected lifetime to develop harmful radiation effects [4–7]. Furthermore, stent evaluation requires contrast-enhanced imaging with iodinated contrast agents, which carries a small but non-negligible risk of contrast nephropathy and allergic reactions. Magnetic resonance imaging (MRI) offers a potential solution for the abovementioned issues, since no ionizing radiation is used and gadolinium, a paramagnetic contrast-agent, is considered relatively safe [8]. The use of MRI has however been limited, since most large vessel stents were made of stainless steel, which leads to MRI susceptibility artifacts hampering in-stent evaluation. New stent materials like platinum are associated with less susceptibility artifacts and may allow for MRI assessment [9,10]. Furthermore, over the past years there has been a shift towards more paediatric-friendly imaging with MRI, for example by reducing the in-bore sound pressure and creating a more comfortable atmosphere [11].

Great vessel stents have been used for almost 25 years to treat congenital heart disease in children. Different stent materials might benefit from certain imaging techniques, however the stent-specific preferred imaging modality for follow-up imaging remains unclear [12–14]. In light of the considerations above, the purpose of this in-vitro study was to evaluate and compare the accuracy of stent lumen quantification in commonly implanted great vessel stents using both CT and MRI.

## Materials and methods

### Stents

**Fig 1:**
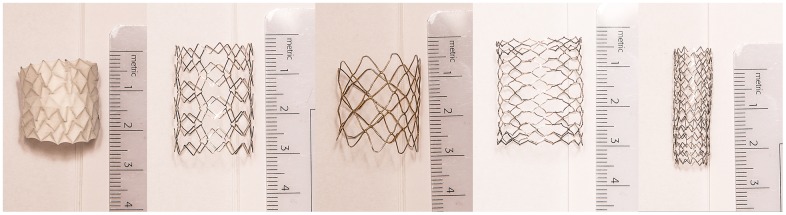
Strut design of different stents. From left to right Atrium V12 covered stent, AndraStent, CP stent, Max LD stent and the Cook Formula stent.

In total, five different great vessel stents were used for this study of which four were inflated to a diameter of 20 mm: Atrium V12 covered stent (316L stainless steel, covering of PTFE), Andramed AS-30 XL (cobalt-chromium), CP stent (0.013” platinum / iridium wire) and EV3 LD Max (stainless steel). The fifth stent, the Cook Formula 535 (316L stainless steel), was inflated to a diameter of 10 mm, as is recommended by the manufacturer. Atlas balloons (Bard inc, USA) with diameters of 10 and 20 mm respectively were inflated with 10 atmospheres in order to achieve the right stent diameters. An overview of the different stents is provided in Table 1 and Fig 1.

**Table 1:** Overview of the evaluated pediatric stents, materials and expanded stent sizes.

Stent	Manufacturer	Material	Size* (mm)
Atrium V12 covered stent	Atrium	316L Stainless steel	20
AndraStent AS30 XL	Andramed GmbH	Cobalt chromium	20
Cheatham Platinum (CP) stent	NuMed Inc.	0.013” platinum / iridium	20
Intrastent Max LD	EV3 Inc	Stainless steel	20
Formula 535 vascular	Cook Medical	316L stainless steel	10

*All stents were expanded to their maximal diameter by inflation of a CBV Balt balloon.

### CT protocol

**Fig 2:**
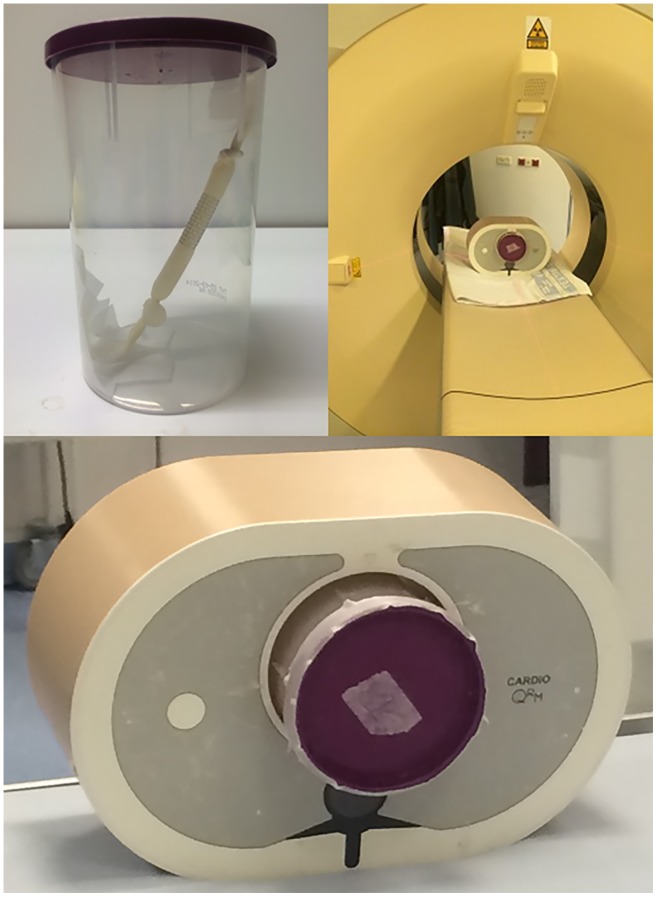
Stent preparation. Each stent containing a balloon with diluted contrast gel was mounted under an angle of 30 degrees in a plastic holder (A). The plastic holder containing the stent and a contrast-gel mixture was put in a commercially available anthropomorphic chest phantom for CT acquisition (B, C).

The stents were placed around a balloon containing diluted contrast-gel to mimic aortic luminal attenuation at CT-angiography. The diluted contrast-gel was a mixture of contrast medium *(Ultravist 300 mg/mL*, *Bayer B*.*V*., *Mijdrecht*, *Netherlands)* and 2% Agar gel solution in a 1:20 ratio. The stents were fixed into a plastic holder under an angle of approximately 30 degrees to simulate the in vivo relation of the aorta to the CT X-ray beam *(*Fig 2*)*. After this, the plastic holder was filled with 2% Agar solution to simulate extravascular CT attenuation. The plastic holder was put into a refrigerator to harden the gel. For CT acquisition, the plastic holder was placed in a commercially available anthropomorphic chest phantom (*QRM GmbH*, *Moehrendorf*, *Germany)* [15] to simulate radiation absorption of a small person. Image acquisition was performed using a 256-slice multidetector-row CT scanner (iCT, Philips Healthcare, Best, The Netherlands) with a collimation of 128 x 0.625 mm and a rotation time of 0.27 seconds. The routine clinical protocol with a tube voltage of 100 kV and tube current-time product of 195 mAs was used with a simulated 60 beats/minute electrocardiogram signal. Reconstructed slice thickness was 0.9 mm. Each stent was scanned eight times to take interscan variation into account. Images were reconstructed with filtered back projection (FBP). The volumetric CT dose index (CTDI_vol_) and dose-length product (DLP) were recorded for each scan. The scan length of the 20 mm stents was 54 mm. For the 10 mm stent a scan length of 73 mm was used. The effective radiation dose was calculated using the conversion factor 0.0235 (100 kV) as recommended for chest CT imaging in a 10-year old [16].

### MRI protocol

**Fig 3:**
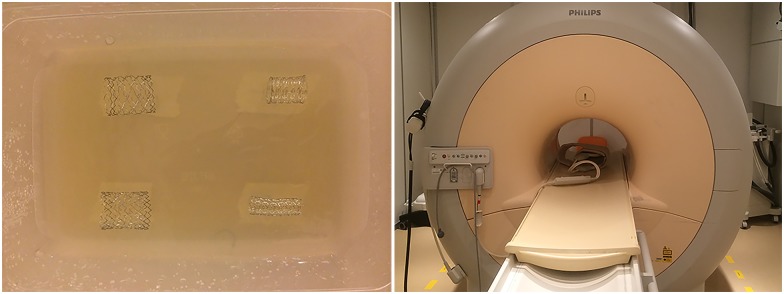
In vitro MRI set up. Example of great vessel stents mounted in diluted paramagnetic contrast gel (A) and placed in the MRI (B).

To create background MRI signal, the stents were placed in a plastic holder containing diluted contrast medium. The mixture consisted of the 1.0 mmol/mL gadolinium based contrast agent gadobutrol (Gadovist, Bayer Healthcare, Berlin, Germany) and 2% Agar solution in a 1:500 ratio. No in-stent balloon was used for MRI. The plastic holder containing the great vessel stents was placed in the SENSE Flex-M coil (Philips Healthcare, Best, The Netherlands) under an angle of approximately 30 degrees *(*Fig 3*)*. Images were acquired using a 1.5 Tesla Achieva MRI scanner (Philips Healthcare, Best, The Netherlands). The following acquisition sequences were used: a multi-slice T_2_-weigthed turbo spin echo, an RF-spoiled three-dimensional T_1_-weighted Fast Field Echo and a balanced turbo field echo 3D. An overview of all MRI sequences and acquisition parameters is provided in S1 Table. The MRI sequences were acquired once.

### Image analysis

Two observers independently obtained the area-derived diameter at the center and both of the outlets of the stent using the circle drawing-tool available in the software in a double-oblique orientation (IntelliSpace Beta, *Philips Healthcare*, *Best*, *The Netherlands*). For CT, images were reconstructed exactly in plane with the stent using the multiplanar reformatting tool. CT measurements were performed using a fixed window width of 2000 Hounsfield Units (HU) and a window level of 800 HU to decrease measurement errors due to metal blooming artifacts. For MRI, no multiplanar reformations were required as images of the stents were acquired in plane with the stent. Window width and window level for MRI measurements were set at the discretion of the observer. In all stents the visible inner diameters were measured by drawing a circle and using the area derived diameter. The circle was drawn as large as possible within the stent lumen. In-stent visibility was defined as the measured stent lumen diameter divided by the true stent diameter (based on the atlas balloon inflation) times 100 percent. Data are provided in S2 Table.

### Statistical analysis

Statistical analysis was performed using IBM SPSS Statistics version 20.0 for Windows. To evaluate the reproducibility of stent diameter measurements the two-way random intraclass correlation coefficient (ICC, consistency) was calculated. For assessment of the interobserver agreement of the measurements, Bland-Altman analyses with 95% limits of agreement were performed. Values are presented as median (IQR) unless stated otherwise.

## Results

The effective radiation dose per CT acquisition was 1.2 mSv (CTDI_vol_ 7.7 mGy, DLP 51.2 mGy*cm).

### Reproducibility

An overview of all results on reliability and agreement for in-stent diameter measurements is provided in Table 2. Overall, interobserver reliability was excellent for both MRI and CT derived diameters. The ICC for MRI ranged from 0.924 to 0.977 depending on the MRI sequence (overall 0.951). With CT, the interobserver ICC was 0.997. Limits of agreement for in-stent diameter measurements were smallest with CT compared with MRI with an overall mean difference of -0.51 mm (95% CI -1.07–0.05) versus -0.05 mm (95% CI -2.52–2.41), respectively. The smallest limits of agreement with MRI were found with the T_2_-weighted turbo spin echo sequence with a mean difference of 0.23 mm (95% CI -1.44–1.91) between real diameter and measured diameter.

**Table 2:** Overall interobserver reliability and agreement for stent diameter measurements *ICC = intraclass correlation coefficient; 95% CI = 95% confidence interval*.

	Reliability (ICC)	Mean difference (95%CI), mm
**MRI (overall)**	0.951	-0.05 (-2.52–2.41)
T_2_-weighted	0.977	0.23 (-1.44–1.91)
T_1_-weighted Fast Field Echo	0.974	-0.80 (-2.66–1.06)
Balanced turbo field echo 3D	0.924	0.40 (-2.65–3.46)
**CT**	0.997	-0.51 (-1.07–0.05)

Interobserver agreement was good for all stent diameters on the CT acquisition with small limits of agreement *(*Table 3*)*. Results for the interobserver agreement per stent per MRI protocol are presented in Table 4. The optimal MRI sequence differed per stent.

**Table 3:** CT interobserver agreement for stent diameter measurements analyzed per stent type. *95% CI = 95% confidence interval*.

	True diameter stent (mm)	Mean difference (95%CI), mm
Atrium V12 Covered	20	-0.50 (-0.89 –-0.12)
Andramed AS30 XL	20	-0.28 (-0.65–0.08)
CP stent	20	-0.48 (-0.98–0.02)
Max LD	20	-0.45 (-1.12–0.21)
Cook Formula 535	10	-0.81 (-1.10 –-0.51)

**Table 4:** MRI interobserver agreement for stent diameter measurements analyzed per stent type. *95% CI = 95% confidence interval*.

		Mean difference (95%CI), mm			
	*True diameter stent (mm)*	*Overall*	*T*_*2*_*-weighted*	*T*_*1*_*-weighted Fast Field Echo*	*Balanced turbo field echo 3D*
Atrium V12 Covered	20	0.20 (-2.79–3.18)	0.27 (-0.86–1.41)*	-0.64 (-2.21–0.93)	0.81 (-4.58–1.41)
Andramed AS30 XL	20	-0.13 (-1.57–1.31)	0.27 (-1.49–2.02)	-0.24 (-1.61–1.12)	0.38 (-0.27–1.04)*
CP stent	20	0.18 (-1.47–1.83)	0.59 (-1.02–2.20)	0.81 (-1.56–3.18)	-0.33 (-1.93–1.28)*
Max LD	20	-0.56 (-4.22–3.10)	-0.47 (-3.49–2.56)	-0.16 (-0.62–0.29)*	0.63 (-3.69–4.95)
Cook Formula 535	10	-0.67 (-2.84–1.50)	0.30 (-0.55–1.15)	0.45 (0.23–0.66)*	0.52 (-2.70–3.74)

* sequence with smallest limits of agreement.

### In-stent visibility

**Fig 4:**
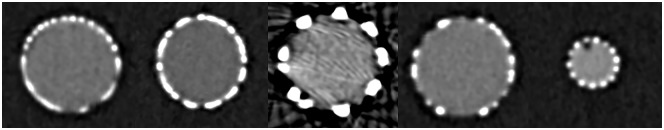
Example of all studied great vessel stents with CT (from left to right) the Atrium V12 covered stent, AndraStent, CP stent, Max LD stent and the Cook Formula stent (10 mm).

**Fig 5:**
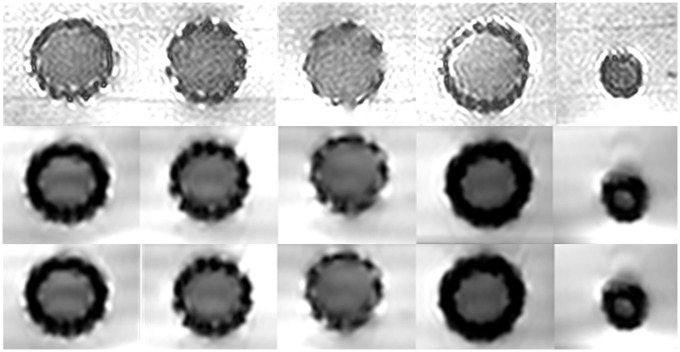
Example of all studied great vessel stents with MRI using T_2_-weighted turbo spin echo (upper), T_1_-weighted Fast Field Echo (middle) and balanced turbo field echo 3D (lower). From left to right Atrium V12 covered stent, AndraStent, CP stent, Max LD stent and the Cook Formula stent (10 mm).

Results with regard to in-stent visibility are provided in Table 5. An example is provided in Figs 4 and 5. In-stent luminal diameters were slightly larger with CT compared to MRI, for all stent types at both the center and the outlets of the stents. Median CT in-stent visibility was 88% (range: 86–90). For MRI, the T_2_-weighted sequence showed the highest lumen visibility (82%; range: 78–84%) compared with T_1_-weighted Fast Field Echo (77%; range: 71–81%) and balanced turbo field echo (74%; range: 65–79%).

**Table 5:** In-stent lumen visibility. Presented are the median percentages for the measured intraluminal stent diameter as proportion of the true stent diameter per acquisition protocol and stent. Optimal stent lumen visibility with MRI was achieved using a T_2_-weighted sequence. *C = center*, *O = outlet*.

	Overall (median [IQR])	Atrium V12 Covered stent		Andramed AS30 XL		CP stent		Max LD		Cook Formula 535	
**MRI**	78 [71–82]	C	O	C	O	C	O	C	O	C	O
T_2_-weighted	82 [78–84]	82	81	79	83	83	87	80	85	75	68
T_1_-weighted Fast Field Echo	77 [71–81]	76	76	80	79	83	86	77	73	60	59
balanced turbo field echo 3D	74 [65–79]	73	79	77	79	71	79	70	69	58	57
**CT**	88 [86–90]	89	89	90	88	89	89	92	93	79	80

Stent lumen visibility for the Atrium V12 stent was good to excellent at the stent center and outlets with both MRI and CT (73%– 89%). The Andramed AS30 XL stent resulted in 90% and 88% lumen visibility with CT at the center and outlets respectively while this was slightly lower with MRI (77–80% and 79–83% respectively). The CP stent provided similar results as the Andramed AS30 XL stent. Stent lumen visibility for the Max LD stent was superior with CT (92% and 93% respectively at the center and outlets) compared to MRI (71–83% and 79–87% respectively). The 10mm Cook Formula 535 stent resulted in the lowest stent lumen visibility of 79% and 80% with CT and 58–75% and 57–68% with MRI respectively at the center and outlets of the stent.

## Discussion

This in vitro study showed that it is possible to achieve good in-stent lumen visibility with both CT and MRI in different pediatric great vessel stents. Overall reliability for stent lumen visibility assessment was good with both CT and MRI. For each stent, interobserver results for diameter measurements differed per MRI sequence. In this study, optimal stent lumen visibility was achieved using a T_2_-weighted sequence for all great vessel stents.

To detect complications like in-stent stenosis and aneurysm formation, follow-up is necessary [2,3]. Recent guidelines recommend both MRI and CT as preferred non-invasive techniques for depicting the aorta, however there are no clear recommendations about the duration and frequency of follow-up [17]. The main concern in patients with aortic stents in daily practice is the radiation exposure associated with CT imaging, especially in children and young patients. With MRI there is no radiation exposure and furthermore, MRI contrast agents are considered to be less toxic than iodine-based CT contrast agents. However, one of the initial concerns with using MRI was that interaction of the magnetic field with the stent could cause heating and dislocation of the stent. Several studies investigated the effect of radiofrequency-induced heating in coronary stents and found no significant heating of the stents [18–20]. Furthermore, the forces caused by the magnetic field on the stent have been shown to be insignificant compared to the physiological forces in the cardiovascular system [19,21]. Therefore, great vessel stents should no longer be considered a contra-indication for MRI acquisition.

Optimal imaging of stents is dependent on stent material, diameter and design [22,23] and many studies have been published on the assessment of coronary artery stents, as these stents are implanted frequently. However, pediatric great vessel stents have a larger diameter and different stent design and are therefore not completely comparable to coronary artery stents. To our best knowledge, only one study investigated imaging with both CT and MRI for great vessels stents. Nordmeyer et al. [9] compared conventional angiography, CT and MRI with three types of great vessel stents (nitinol, platinum-iridium, stainless steel). Three lumen conditions were simulated, namely no stenosis, internal stenosis and external stenosis to assess the sensitivity and specificity of CT and MRI with conventional angiography as the gold standard. Sensitivity and specificity was high for both CT and MRI. However, in the Nordmeyer study quantitative assessment of the stent lumen was inaccurate with MRI in the stainless steel stent while CT showed good agreement with angiography in all stents. In the present study, however, even stainless steel stents showed acceptable limits of agreement for stent diameter measurements, although results were dependent on the MRI sequence used. Another study performed by Eichhorn and colleagues [24] compared conventional angiography to CT both in vitro and in patients. Two CT protocols were used with an effective dose of 1.8 and 0.6 mSv respectively. There were no differences in image quality between those protocols. There was good agreement between angiography and CT both in vitro and in patients, however mild stenosis was missed or underestimated with CT. Mild stenosis was defined as 25% lumen narrowing in vitro, and subjectively assessed in patients. Also, stent lumen was underestimated with CT and stent struts appeared significantly thicker on CT compared to conventional angiography. These findings are comparable to the current study. Although we did not compare the results to conventional angiography, we also found that the stent lumen was underestimated with CT, which is caused by blooming artifacts.

This study has several limitations. First, it concerns an in vitro study. Therefore, the influence of motion artifacts remains unknown. However, due to the in vitro set up we were able to investigate different CT and MRI protocols that can be used to set up a patient study. Second, one stent had a smaller diameter which might limit the comparability to the other stents. Since this was the maximal vendor recommended diameter, it was not dilated to a higher diameter. Future studies should determine the influence of stent diameter by inflating the same stent to different sizes. Third, MRI sequences were only acquired once for practical reasons, while the CT acquisitions were repeated eight times. Fourth, we did not investigate diagnostic accuracy of CT and MRI for stenosis detection.

In conclusion, stent lumen visibility is most accurately measured with CT and the T_2_ weighted sequence on MRI. Both with CT and MRI true stent diameter is underestimated by approximately 20%. Furthermore stent lumen visibility can be measured with excellent interobserver reliability on both CT and all MRI sequences. However, common conditions such as in-stent stenosis and associated aneurysms were not tested in this in-vitro study, limiting the value of the in-vitro study.

## Supporting information

S1 Table MRI acquisition parameters. (DOCX)

S2 Table Data. This table contains all measurements for the different stent types with both MRI and CT. Measurements were performed at three locations, namely at the two outlets and at the center of the stent.
(PDF)
