# Supplementary material for: Imaging of pediatric great vessel stents: Computed tomography or magnetic resonance imaging?
Source: PLoS One. 2017 Jan 31;12(1):e0171138. doi: 10.1371/journal.pone.0171138 (PMC5283725; doi:10.1371/journal.pone.0171138)
Supplement: S1 Table — (DOCX) [file pone.0171138.s001.docx]

**Supporting Information**

**S1 Table - MRI acquisition parameters**

|  | T_2_-weighted | T_1_-weighted Fast Field Echo | Balanced turbo field echo 3D |
| --- | --- | --- | --- |
| Repetition Time (ms) | 3000 | 5 | 3 |
| Echo Time (ms) | 80 | 2 | 1 |
| Echo Train | 20 | 1 | 34 |
| Flip angle (degrees) | 90 | 20 | 45 |
| Slice thickness (mm) | 2.50 | 3.00 | 4.00 |
| Matrix size | 240 | 350 | 163 |
| FOV | 640x640 | 640x640 | 256x256 |
