# Supplementary material for: Imaging of pediatric great vessel stents: Computed tomography or magnetic resonance imaging?
Source: PLoS One. 2017 Jan 31;12(1):e0171138. doi: 10.1371/journal.pone.0171138 (PMC5283725; doi:10.1371/journal.pone.0171138)
Supplement: S2 Table — This table contains all measurements for the different stent types with both MRI and CT. Measurements were performed at three locations, namely at the two outlets and at the center of the stent. (PDF) [file pone.0171138.s002.pdf]

Location	Acquisition	Atrium V12 Covered stent, diameter (mm) - rater 1	Atrium V12 Covered stent, diameter (mm) - rater 2	Andramed AS30XL stent, diameter (mm) - rater 1	Andramed AS30XL stent, diameter (mm) - rater 2	CP stent, diameter (mm) - rater 1	CP stent, diameter (mm) - rater 2
Outlet (1)	T2-weighted	16,81	16,03	16,54	16,73	17,30	17,10
Outlet (1)	T1-weighted Fast Field I	15,17	15,77	15,06	16,18	16,47	17,36
Outlet (1)	Balanced Turbo Field Ec	16,51	15,90	15,90	15,64	15,64	15,64
Center	T2-weighted	16,60	16,20	16,41	15,11	17,19	15,81
Center	T1-weighted Fast Field I	15,17	15,37	15,87	16,09	16,61	16,47
Center	Balanced Turbo Field Ec	16,51	12,85	15,39	15,26	14,38	14,10
Outlet (2)	T2-weighted	15,67	16,03	16,42	16,73	18,07	17,26
Outlet (2)	T1-weighted Fast Field I	14,67	15,37	15,57	16,30	17,85	16,91
Outlet (2)	Balanced Turbo Field Ec	14,38	16,21	16,02	15,26	15,13	16,39
Outlet (1)	CT (acquisition 1)	17,78	18,10	17,67	17,93	17,70	18,02
Outlet (1)	CT (acquisition 2)	17,73	18,00	17,61	17,97	17,78	18,03
Outlet (1)	CT (acquisition 3)	17,65	18,05	17,67	17,95	17,79	17,93
Outlet (1)	CT (acquisition 4)	17,71	18,05	17,63	17,95	17,79	17,95
Outlet (1)	CT (acquisition 5)	17,78	18,04	17,63	17,96	17,81	17,97
Outlet (1)	CT (acquisition 6)	17,77	18,03	17,68	17,99	17,74	18,00
Outlet (1)	CT (acquisition 7)	17,74	18,13	17,57	17,95	17,86	17,95
Outlet (1)	CT (acquisition 8)	17,50	18,09	17,61	17,95	17,64	17,92
Center	CT (acquisition 1)	17,48	17,99	17,99	18,02	17,43	18,03
Center	CT (acquisition 2)	17,47	17,95	18,03	18,11	17,48	17,96
Center	CT (acquisition 3)	17,48	17,99	17,99	18,11	17,53	17,99
Center	CT (acquisition 4)	17,44	17,90	17,96	18,09	17,52	17,90
Center	CT (acquisition 5)	17,45	17,98	18,07	18,08	17,69	17,95
Center	CT (acquisition 6)	17,51	17,95	18,09	18,06	17,50	17,94
Center	CT (acquisition 7)	17,54	17,89	18,06	18,10	17,45	17,95
Center	CT (acquisition 8)	17,35	17,96	18,04	18,13	17,51	17,98
Outlet (2)	CT (acquisition 1)	17,00	18,02	17,23	17,74	17,28	18,00
Outlet (2)	CT (acquisition 2)	17,08	17,98	17,07	17,67	17,23	18,06
Outlet (2)	CT (acquisition 3)	17,17	17,99	17,23	17,71	17,25	17,96
Outlet (2)	CT (acquisition 4)	17,42	17,96	17,13	17,72	17,29	17,98
Outlet (2)	CT (acquisition 5)	17,47	17,98	17,23	17,68	17,14	17,94
Outlet (2)	CT (acquisition 6)	17,61	17,99	17,19	17,60	17,25	18,00
Outlet (2)	CT (acquisition 7)	17,47	17,99	17,19	17,61	17,20	18,02
Outlet (2)	CT (acquisition 8)	17,36	18,05	17,23	17,62	16,98	17,93

Location	Acquisition	Max LD stent, diameter (mm) - rater 1	Max LD stent, diameter (mm) - rater 2	Cook Formula 535 stent, diameter (mm) - rater 1	Cook Formula 535 stent, diameter (mm) - rater 2
Outlet (1)	T2-weighted	15,81	18,00	6,95	6,70
Outlet (1)	T1-weighted Fast Field Echo	12,28	15,77	5,36	6,36
Outlet (1)	Balanced Turbo Field Echo 3D	15,39	12,22	6,46	5,09
Center	T2-weighted	16,03	16,03	7,62	7,29
Center	T1-weighted Fast Field Echo	14,93	15,72	5,64	6,36
Center	Balanced Turbo Field Echo 3D	13,60	14,38	5,09	6,46
Outlet (2)	T2-weighted	17,30	16,51	6,95	6,63
Outlet (2)	T1-weighted Fast Field Echo	14,49	15,77	5,36	6,71
Outlet (2)	Balanced Turbo Field Echo 3D	13,60	14,10	6,46	4,89
Outlet (1)	CT (acquisition 1)	18,96	19,03	7,49	8,49
Outlet (1)	CT (acquisition 2)	18,88	18,96	7,42	8,43
Outlet (1)	CT (acquisition 3)	18,92	19,03	7,52	8,51
Outlet (1)	CT (acquisition 4)	18,88	18,99	7,58	8,49
Outlet (1)	CT (acquisition 5)	18,68	18,96	7,62	8,44
Outlet (1)	CT (acquisition 6)	18,83	18,93	7,62	8,44
Outlet (1)	CT (acquisition 7)	18,79	19,04	7,61	8,51
Outlet (1)	CT (acquisition 8)	18,70	18,96	7,52	8,47
Center	CT (acquisition 1)	18,28	18,60	7,58	8,27
Center	CT (acquisition 2)	18,21	18,63	7,55	8,24
Center	CT (acquisition 3)	18,26	18,63	7,58	8,25
Center	CT (acquisition 4)	18,42	18,55	7,63	8,28
Center	CT (acquisition 5)	18,33	18,59	7,77	8,26
Center	CT (acquisition 6)	18,33	18,56	7,60	8,21
Center	CT (acquisition 7)	18,33	18,63	7,68	8,26
Center	CT (acquisition 8)	18,19	18,64	7,65	8,23
Outlet (2)	CT (acquisition 1)	17,76	18,62	7,40	8,36
Outlet (2)	CT (acquisition 2)	17,71	18,54	7,44	8,40
Outlet (2)	CT (acquisition 3)	17,74	18,62	7,52	8,32
Outlet (2)	CT (acquisition 4)	17,63	18,63	7,42	8,29
Outlet (2)	CT (acquisition 5)	17,58	18,54	7,49	8,36
Outlet (2)	CT (acquisition 6)	17,66	18,62	7,48	8,33
Outlet (2)	CT (acquisition 7)	17,73	18,60	7,55	8,29
Outlet (2)	CT (acquisition 8)	17,72	18,61	7,44	8,35
